# Molecular mediators of peritoneal metastasis in pancreatic cancer

**DOI:** 10.1007/s10555-020-09924-4

**Published:** 2020-08-11

**Authors:** Leela Rani Avula, Brendan Hagerty, Christine Alewine

**Affiliations:** 1 Laboratory of Molecular Biology, National Cancer Institute, National Institutes of Health, Bethesda, MD USA; 2 Surgical Oncology Program, National Cancer Institute, National Institutes of Health, Bethesda, MD USA

**Keywords:** Peritoneal metastasis, Pancreatic adenocarcinoma, Gastric cancer, Ovarian cancer

## Abstract

Pancreatic cancer is the third leading cause of cancer death in the USA, and pancreatic ductal adenocarcinoma (PDA) constitutes 85% of pancreatic cancer diagnoses. PDA frequently metastasizes to the peritoneum, but effective treatment of peritoneal metastasis remains a clinical challenge. Despite this unmet need, understanding of the biological mechanisms that contribute to development and progression of PDA peritoneal metastasis is sparse. By contrast, a vast number of studies have investigated mechanisms of peritoneal metastasis in ovarian and gastric cancers. Here, we contrast similarities and differences between peritoneal metastasis in PDA as compared with those in gastric and ovarian cancer by outlining molecular mediators involved in each step of the peritoneal metastasis cascade. This review aims to provide mechanistic insights that could be translated into effective targeted therapies for patients with peritoneal metastasis from PDA.

Pancreatic cancer is an intractable malignancy and the third leading cause of cancer death in the USA. Although it constitutes a small percentage of all cancer deaths around the world (4.5%), it is one of the most fatal types of cancer with an overall median 5-year survival rate of about 10% [1–3]. The most common histology of pancreatic cancer, contributing to 85% of all diagnoses, is pancreatic ductal adenocarcinoma (PDA), which arises in the exocrine glands of the organ [1, 2]. PDA represents a challenge for clinicians in terms of early detection and management. No reliable biomarkers for early detection have been identified so far [4]. Lack in early detection can also be attributed to the relatively silent progression and nonspecific symptom presentation of pancreatic tumors at early stages. Most patients present with local disease too advanced for surgical resection or with their tumor already spread to distant organs [5–9]. Less than one in five patients has early stage disease suitable to undergo potentially curative resection, and of those only 20% survive 5 years [9]. Cure rates following resection, which have historically failed to reach 10%, are now modestly improving with the advent of new combination chemotherapy regimens such as FOLFIRINOX. However, even cures are accompanied by a high incidence of toxic side effects that negatively impact patient quality of life [10–12]. PDA’s incidence and mortality rates have been increasing for decades, and it is expected to become the second leading cause of cancer-related death in the USA by 2030 [13]. Nevertheless, advances in research and healthcare delivery have produced a steady increase in 5-year median overall survival through the last decade [1, 14].

One of the main metastatic pathways of PDA is peritoneal dissemination. Other common sites of PDA metastasis include the liver, lungs and pleura, bones, and adrenal glands [6, 15, 16]. Peritoneum is the 2nd most common metastatic site after liver, and peritoneal metastases are present in 50% of patients with pancreatic cancer at the time of death [15, 16]. Approximately 9% of PDA cases already have established peritoneal metastases at the time of diagnosis [8]. In a retrospective study of autopsied patients who died of recurrence following potentially curative resection of PDA, one third were reported to have developed peritoneal dissemination [17]. Other retrospective studies showed that about 25–50% of the patients who died from PDA with or without systemic treatment or surgery had peritoneal metastases [6, 16, 18]. While the tumor histology is an important factor, the anatomical location of the primary tumor may also be a key determinant in the development of peritoneal metastasis [15, 19, 20]. The tail region of the pancreas is located intraperitoneally, and the rest of the organ is positioned retroperitoneally, deep within the upper abdomen in the epigastrium and left hypochodrium regions. During PDA progression, cancer cells released from the surface of the tumor can adhere to and invade tissues and organs in the peritoneal cavity. In some cases, the detached cells directly penetrate the peritoneal cavity from the primary site [15, 16, 21]. Malignant cells from PDA are shed into the peritoneum early and commonly, and their presence seems to be indicative of an adverse prognosis [22]. Such cells are found within the peritoneal cavity in 20–30% of patients with early-stage disease undergoing potentially curative resection who otherwise have no peritoneal or liver metastases [16]. The presence of peritoneal micrometastases, even in the absence of macroscopic metastases, also has a dismal outcome, and such patients do not benefit from local treatments including irradiation or surgery [23]. The dispersed growth pattern of peritoneal metastases makes their identification difficult on imaging studies, and thus direct visualization, either at laparotomy or through laparoscopy, is frequently required to determine the extent of disease [16, 24]. The prognosis for patients with PDA that has spread to the peritoneum is poor, and peritoneal disease is an important cause of morbidity and mortality [8, 25]. Peritoneal metastasis is a difficult problem in PDA.

The current treatment options for pancreatic peritoneal carcinomatosis include chemotherapy and palliative care. Systemic combination chemotherapy with FOLFIRINOX or gemcitabine/nab-paclitaxel can be effective for peritoneal metastasis just as it is for disease elsewhere [26]. Nevertheless, effective palliation of PDA peritoneal metastasis remains a challenge for physicians: patients are frequently troubled by abdominal pain, poor gastrointestinal (GI) motility, malignant ascites, and bowel obstruction which negatively impacts their quality of life and accelerates cancer-associated weight loss [8, 27]. A large study in the Netherlands showed that patients with PDA peritoneal carcinomatosis who are not undergoing systemic treatment have a median overall survival of just 6 weeks after diagnosis [8]. Other studies in pancreatic cancer patients with peritoneal metastasis have shown a median survival of less than 3 months if left untreated [28, 29]. When treated with combination chemotherapy, patients with peritoneal metastasis from pancreatic cancer have a median overall survival of about 8 months [25, 28]. Peritoneal carcinomatosis of ovarian and non-PDA GI cancers can be treated with locoregional intraperitoneal chemotherapy and cytoreductive surgery with promising results [8, 30]. However, using similar treatment strategies in patients with peritoneal carcinomatosis of PDA origin has not resulted in significant benefit for patients in terms of survival or quality of life. Trials investigating treatment strategies for peritoneal carcinomatosis rarely include patients with PDA pathology. Trials exclusively focused on peritoneal carcinomatosis of PDA origin are even more sparse. Furthermore, most trials include very few PDA patients and often do not have a comparator arm. The limited available data suggests that no locoregional approach provides meaningful survival benefit for these patients. Results of therapeutic trials directed at peritoneal carcinomatosis including PDA patients are summarized in Table 1 [31–41]. Ongoing trials without published results are summarized in Table 2.

**Table 1:** Published trials and case series of patients treated with locoregional therapy for peritoneal carcinomatosis from PDAC

Author	Year	Title	*n*^a^	PDA outcomes
Budd [31]	1986	Phase I trial of intraperitoneal chemotherapy with 5-fluorouracil and citrovorum factor	1	Objective PR
Lenzi [32]	2002	Phase I study of intraperitoneal recombinant human interleukin 12 in patients with Müllerian carcinoma, gastrointestinal primary malignancies, and mesothelioma	1	PD
Morgan [33]	2003	Phase I trial of intraperitoneal docetaxel in the treatment of advanced malignancies primarily confined to the peritoneal cavity	1	Not reported
Farma [34]	2005	Limited survival in patients with carcinomatosis from foregut malignancies after cytoreduction and continuous hyperthermic peritoneal infusion	7	2–62 months OS
Strohlein [35]	2011	Immunotherapy of peritoneal carcinomatosis with the antibody catumaxomab in colon, gastric, or pancreatic cancer: an open-label, multicenter, phase I/II trial	3	2–9 months OS
Ishikawa [36]	2012	Phase II trial of combined regional hyperthermia and gemcitabine for locally advanced or metastatic pancreatic cancer	2	5 months median OS^b^
Takahara [37]	2016	Intravenous and intraperitoneal paclitaxel with S-1 for treatment of refractory pancreatic cancer with malignant ascites	35	4.8 months median OS
Graverson [38]	2017	Peritoneal metastasis from pancreatic cancer treated with pressurized intraperitoneal aerosol chemotherapy (PIPAC)	5	3 alive at time of report^c^
Khosrawipour [39]	2017	Pressurized intra peritoneal aerosol chemotherapy in patients suffering from peritoneal carcinomatosis of pancreatic adenocarcinoma	20	36 weeks median OS
Satoi [40]	2017	Multicenter phase II study of intravenous and intraperitoneal paclitaxel with S-1 for pancreatic ductal adenocarcinoma patients with peritoneal metastasis	33	Median OS 16 months
Tentes [41]	2018	Cytoreduction and HIPEC for peritoneal carcinomatosis of pancreatic cancer	6	1–36 months OS

*PR*, partial response; *PD*, progressive disease; *OS*, overall survival

^a^Patients in study with PC due to PDAC

^b^For entire cohort of metastatic patients, not limited to PC

^c^Median 6 months after first treatment

**Table 2:** Ongoing or completed clinical trials for peritoneal carcinomatosis from PDAC

PI	NCT No.	Title	Status
Sciotto	04000906	PIPAC with Nab-paclitaxel and cisplatin in peritoneal carcinomatosis (Nab-PIPAC)	Not yet recruiting
Katz	03682744	CAR-T intraperitoneal infusions for CEA-expressing adenocarcinoma peritoneal metastases or malignant ascites (IPC)	Active, not recruiting
Bartlett	02151448	αDC1 vaccine + chemokine modulatory regimen (CKM) as adjuvant treatment of peritoneal surface malignancies	Completed
Lenzi	00003046	Interleukin-12 in treating patients with cancer in the abdomen	Completed
Ceelen	03304210	PIPAC Nab-pac for stomach, pancreas, breast and ovarian cancer (PIPAC-nabpac)	Recruiting
Topal	01116791	Cytoreductive surgery (CRS) plus hyperthermic intraoperative peritoneal chemotherapy (HIPEC) with cisplatin to treat peritoneal carcinomatosis from upper gastrointestinal cancer	Terminated
Meredith	01384253	Safety study of ^212^Pb-TCMC-trastuzumab radio immunotherapy	Completed
Johnson et al	00666991	Pharmacokinetic, safety and efficacy study of nanoparticle paclitaxel in patients with peritoneal cancers	Completed

Despite the significance of peritoneal carcinomatosis in PDA, the mechanisms underlying this metastatic process remain poorly understood. In contrast with the vast body of research that has addressed hematogenous metastasis leading to hepatic or pulmonary spread in PDA, fewer studies have investigated the biology of peritoneal dissemination. While hematogenous metastasis occurs through the systemic circulation, the peritoneal metastasis process occurs mainly by exfoliation of cells from the tumor into the peritoneal fluid of the intrabdominal cavity. The free-floating cancer cells then invade into peritoneal surfaces [21]. This review aims to describe mechanisms of peritoneal carcinomatosis which are important in PDA by examining the existing literature on mechanisms of PDA peritoneal spread, and through correlation with what is known about ovarian and gastric cancer peritoneal metastasis.

## Pancreatic cancer hematogenous and peritoneal metastasis are distinct processes

Unsurprisingly, the factors required for passive metastasis in the peritoneal cavity are very different from those necessary for hematogenous spread, where tumor cells are exposed to the hydrodynamic forces present during rapid transport through the blood vessels [21]. In fact, Nishimori et al. [42, 43] showed that cells optimized for peritoneal dissemination have no observed advantage in hepatic colonization. Global gene expression and molecular profiling studies comparing PDA-derived hematogenous metastasis *versus* peritoneal dissemination employing optimized metastatic cell lines and animal models identified key differentiating factors. Some of these factors include angiogenesis protein angiopoietin 1, extracellular matrix (ECM) protease, matrix metalloproteinase 10 (MMP10), cytokine IL-8, integrin-binding protein osteopontin, hepatocyte growth factor (HGF), vascular endothelial growth factor (VEGF), tumor suppressor/apoptosis genes, regulatory transcription factors, cell adhesion proteins, and membrane receptors [42–49]. These data demonstrate that considerable mechanistic differences underly PDA-derived hematogenous metastasis *versus* peritoneal dissemination and suggest that therapies specific to disrupting the latter process may be distinct.

## Methods of spread of peritoneal metastasis

Peritoneal metastasis (also known as transcoelomic metastasis or peritoneal carcinomatosis/dissemination/seeding) involves the spread of malignant tumor throughout the peritoneal cavity and onto the outer surfaces of abdominal and pelvic organs. This process occurs extensively in non-PDA GI cancers (such as gastric and colon cancers) and in ovarian cancers by one of three mechanisms: (i) Single cell or clumps of tumor cells lose cell-cell contact from the primary site and exfoliate into the peritoneal cavity. They are then transported throughout the cavity by peritoneal fluid before seeding peritoneal surfaces. (ii) Seeding occurs, preferentially in the omental milky spots, as a manifestation of systemic metastasis by following the capillary or lymphatic route arising from the primary cancer site. (iii) Accidental peritoneal seeding happens during surgical manipulation through handling of cancer-contaminated blood and lymph channels or by spilling of tumor cells during resection [19, 50, 51]. In addition to the above-mentioned three mechanisms, formation of intraperitoneal metastases is also thought to occur *via* the hematogenous route along the blood vessels rather than peritoneal seeding [52, 53].

Evidence suggests that PDA can use at least two of these mechanisms of peritoneal spread. Transcoelomic dissemination is defined as spread through the vascular network and lymphatic pathway *via* stomata in the diaphragm and milky spots in the omentum and has been observed in PDA [54–56]. Passive peritoneal dissemination in PDA is more common and a relatively gradual process compared with lymphatic absorption [49]. There is no evidence for iatrogenic PDA seeding during surgical or diagnostic procedures, including preoperative EUS-guided FNA (EUS-FNA) [57].

## Tumor genetic changes associated with peritoneal metastasis

In ovarian cancer, several studies identified gene expression patterns important to the biology and treatment response of metastatic disease which is most frequently localized to the peritoneal cavity. For example, Verhaak et al. [58] developed a 100 gene signature for molecular subtyping and characterizing advanced ovarian cancer specimens with peritoneal metastases [58]. Others have used gene expression patterns to characterize relevant molecular pathways operative in the transition of primary ovarian tumors to peritoneal metastases. For example, Brodsky et al. found that peritoneally disseminating cells originating from ovarian cancer were more proliferative and less apoptotic than their respective primary tumors. In addition, peritoneal metastases had copy number aberrations that differed from those found in the primary tumor. They identified a six-gene expression signature to distinguish primary from metastatic tumors [59]. In a similar study of epithelial ovarian cancers, microarray analysis identified a 56 gene set with differential expression contrasting the primary tumor from peritoneal metastases [60]. Of note, 10/56 genes were associated with the p53 gene pathway [60]. Matte et al. studied gene expression changes in human peritoneal mesothelial cells (HPMCs) exposed to peritoneally disseminated ovarian cancer cells derived from ascites [61]. Ascites is the excess fluid that builds up in the abdomen of many cancer patients with peritoneal metastases. A total of 649 genes were differentially expressed in ascites-stimulated HPMCs with 484 genes upregulated and 165 genes downregulated [61]. These findings support the importance of the interplay between cancer cells and HPMCs and define the role that the tumor environment (TME) plays in these interactions. Malek et al. showed that gene expression profiles in peritoneal metastasis are significantly different than their matched primary tumor and that these changes are affected by underlying copy number variation [62]. The differentially expressed genes are enriched in specific pathways including JAK/STAT pathway, cytokine signaling, and other immune-related pathways [62].

Peritoneal metastasis is the most important prognostic factor in gastric cancer and is often associated with a high mortality rate [63]. Differential gene expression profiles of gastric cancer cells with high metastatic potential to peritoneum or those from malignant ascites *versus* primary tumor have been reported [64, 65]. Twenty-four genes involved in cell adhesion, epithelial markers, drug metabolism, and signal transduction were upregulated and 17 genes of immune response, cell cycle, and adhesion were downregulated [65]. Wu et al. uncovered translationally upregulated genes in the context of epithelial to mesenchymal transition (EMT) using polysome profiling and found that six-transmembrane epithelial antigen of the prostate 1 (*STEAP1*) is induced translationally and its expression promotes proliferation, migration, invasiveness, and tumorigenicity of gastric cancer [66]. Another similar cell line study revealed that recombinant human S100 calcium-binding protein A4 (*S100A4*) and cadherin-associated protein β1 (*CTNNB1*) were upregulated while phosphatase and tensin homolog deleted on chromosome tEN (*PTEN*) was downregulated in peritoneally disseminating cells [67]. Chen et al. investigated molecular profiles and metastasis markers in Chinese patients with gastric carcinoma and unraveled mutation spectra and genomic regions associated with peritoneal metastasis. Mutations in microtubule actin crosslinking factor 1 (*MACF1*), cell division cycle 27 (*CDC27*), hemicentin 1 (*HMCN1*), cadherin 1 (*CDH1*), and PDZ domain containing 2 (*PDZD2*) were moderately enriched in peritoneal metastasis samples [68]. Others have also examined the unique mutational landscape, copy number alteration, and gene expression profile of gastric cancer peritoneal metastasis cells [69].

**Fig. 1:**
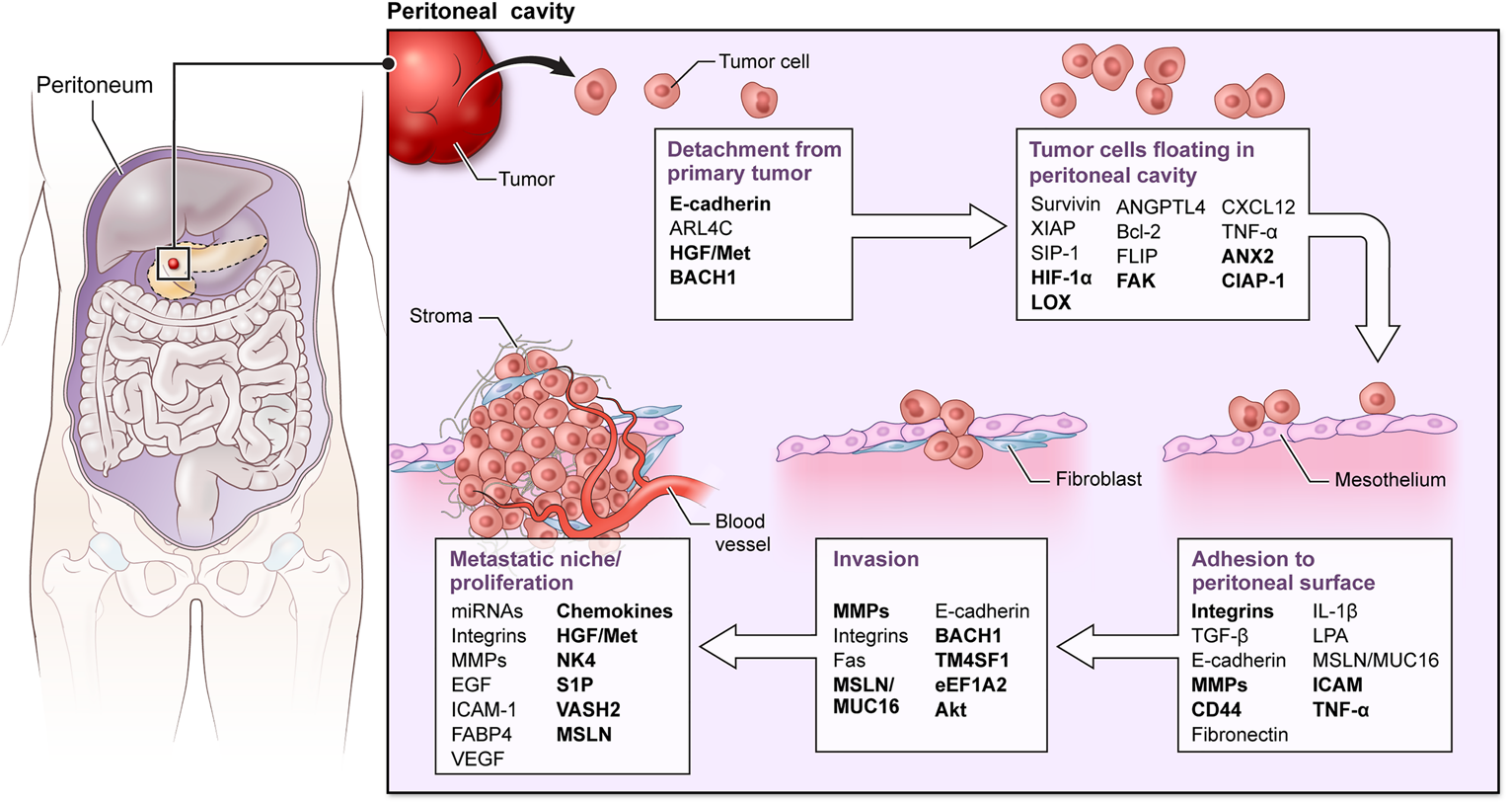
The metastatic cascade of peritoneal metastasis showing the major molecular factors involved in each step of the cascade. Molecules bolded indicate the ones investigated and playing a role in PDA peritoneal metastasis

Although several genes have been shown to be associated with peritoneal metastasis in the tumor types mentioned above, few of these have been functionally assigned to promote specific steps in the peritoneal metastasis cascade (Fig. 1).

Genes that are functionally associated with peritoneal metastasis in ovarian and gastric cancers have been identified to play a role in PDA peritoneal metastasis (Table 3; Fig. 1). To gain insight into the metastatic process used by PDA, Van den Broeck et al. compared gene expression profiles of liver and peritoneal metastases with those of primary tumor samples employing fresh human samples of both liver and peritoneal metastases [139]. Their analysis revealed 29 genes potentially contributing to the metastatic process including *β-catenin* which plays role in cell adhesion/EMT, acidic nuclear phosphoprotein 32 family member A (*ANP32A*) which is a tumor suppressor, 15-hydroxyprostaglandin dehydrogenase (*HPGD*), SET nuclear proto-oncogene (*SET*), and Sp1 transcription factor (*SP1*) [139]. Nomura et al. generated an optimized peritoneally disseminating cell line and compared the expression profile with the parental line. They identified 37 differentially regulated genes which mainly included tumor suppressor/apoptosis genes such as DNA fragmentation factor (*DFF-45*/*ICAD*) and lamin A, and cell adhesion genes such as E-cadherin, Fbn-1 (fibrillin 1), laminin gamma-2 (laminin 1), and vinculin [44].

**Table 3:** The major molecular factors involved in the development of peritoneal metastasis

Steps of peritoneal metastasis	Molecular factors involved	Ovarian cancer peritoneal metastasis	Gastric cancer peritoneal metastasis	PDA peritoneal metastasis
Cell detachment from the primary tumor	E-cadherin	[66]	[65]	[70]
	ARL4C	[71]	[72]	
	HGF/Met	[73]	[74]	[75]
	BACH1	[76]		[77]
Cell survival and transport in the peritoneal fluid	Survivin/XIAP	[78–80]		
	HIF-1α	[81]	[42, 82]	[83]
	LOX	[84]	[84]	[85]
	ANGPTL4		[86]	
	FAK	[87]	[87]	[88]
	CXCL12	[89]	[90]	
	TNF-α	[91]		
	ANX2	[92]		[93]
	CIAP-1			[94]
Attachment to the peritoneal mesothelial cells	Integrins	[95–98]		[33, 34]
	TGF-β		[99]	
	Fibronectin	[100]		
	E-cadherin	[96]		[70]
	MMPs	[97, 98]	[101]	
	CD44	[102]	[103]	[33, 34, 104]
	IL-1β	[105]		[104]
	LPA	[106]		
	MSLN/MUC16	[107]	[108]	
	ICAM-1	[109]	[110]	[104, 111]
	TNF-α	[112]	[113]	[104]
Cell invasion to the underlying connective tissue	MMPs	[102, 114, 115]	[116]	[117, 118]
	Integrins	[114]	[116]	
	Fas	[115]		
	MSLN/MUC16			[117, 119]
	E-Cadherin			[70]
	BACH1	[76]		[77]
	TM4SF1	[120]		[121]
	eEF1A2			[118]
	Akt		[122]	[118]
Establishment of metastatic deposits	miRNAS	[123, 124]	[125]	
	Integrins	[126]		
	MMPs		[122]	
	EGF	[126]		
	ICAM-1	[126]		
	FABP4	[127]		
	VEGF	[126]	[128, 129]	
	Chemokines	[123]		[130]
	PAR-1	[131]	[132]	
	HGF/Met			[75]
	NK4	[133]	[134]	[135]
	S1P			[136]
	VASH2	[137]		[138]
	MSLN		[108]	[119]

To sum up, although individual molecules may be different, the classes of proteins important for peritoneal metastasis process seem to be conserved across the different tumor types. For example, genes involved in cell adhesion, EMT, apoptosis, and tumor suppression all participate regardless of differences in tumor histology (Table 3; Fig. 1).

## Steps of the peritoneal metastasis cascade

The peritoneal metastatic cascade has been well described as a complex multistage process which requires detachment, flotation, adhesion, invasion of tumor cells, and establishment of metastatic deposits [21, 50, 72]. Many metastasis-related factors such as adhesion molecules, ECM components, matrix proteases, and motility factors are involved in the development of peritoneal metastasis. During the process, the following occur sequentially: (1) Malignant cells exfoliate/detach/shed from the primary tumor and disseminate/spread/float through a transcoelomic mechanism following the peritoneal fluid cycles in the peritoneal cavity. In some cases, peritoneal dissemination can also be initially driven by direct invasion from primary site into the peritoneal cavity [21]. (2) Circulating malignant cells surviving in the microenvironment adhere/attach to peritoneal mesothelial cells (PMCs). Multilevel reactions among molecular and cellular components of the malignant cells and the peritoneum dictate the fate of the flowing malignant cells. PMCs provide a surface upon which the invading malignant cells and associated stromal components can adhere. In anchorage-dependent cells such as epithelial cells, loss of this adhesion induces a form of programed cell death, called *anoikis*. Therefore, anoikis resistance is required for cells to survive flotation in the peritoneal cavity and for anchorage-independent growth [71]. (3) Next, cancer cells invade/penetrate to underlying connective tissue. (4) Invasion stimulates sub-mesothelial connective tissues to form a metastatic niche for seeding and establishing metastatic deposits [21, 50, 72]. Neoangiogenesis is required to support this process. In the following sections, we describe the molecular and cellular mechanisms known to regulate each step of the peritoneal metastatic cascade (Table 3; Fig. 1).

## Molecular mediators of cell detachment from the primary tumor

The cell detachment process begins with loosening of the cell-cell attachment at the primary tumor site so that cells become motile and exfoliate [19]. This process is promoted by mechanical forces like rubbing of neighboring peritoneal organs and the natural flow of the peritoneal fluid. An important molecule that helps ovarian and gastric cancer cells to detach and become motile is E-cadherin, a membrane glycoprotein that mediates adherens junction formation between homophilic cells [64, 73, 74, 140]. E-cadherin expression in detached ovarian and gastric cancer cells is lower than that in primary tumor cells. Moreover, attenuation of E-cadherin expression in ovarian and gastric peritoneal carcinomatosis is associated with poor patient survival [141, 142]. Change in E-cadherin expression drives cell motility through activation of Rho GTPases and reorganization of the actin cytoskeleton [70, 76, 77]. Other downstream factors of epidermal growth factor (EGF) pathway signaling and Wingless-related iNTegration site (Wnt) signaling such as ADP-ribosylation factor-like 4C (ARL4C) can also activate Rho GTPases and promote actin cytoskeleton reorganization and cell motility [75, 143]. ARL4C is therefore proposed to be a novel biomarker and potential therapeutic target for ovarian and gastric cancer patients with peritoneal metastasis [75, 144]. HGF and its receptor Met proto-oncogene receptor tyrosine kinase (c-Met) are implicated in the peritoneal metastasis of ovarian and gastric cancer [145, 146]. HGF signaling plays an important role in tumor growth by activating mitogenic signaling pathways. Targeting c-Met *in vivo* using RNAi inhibits peritoneal dissemination through an α_3_β_1_ integrin-dependent mechanism [145, 146]. Cell detachment occurs when integrins located on cancer cells release from their binding partners in the surrounding ECM. Alterations in integrin-mediated ECM-ligand binding can result in decreased cell adhesion, changes in cell morphology, increased migration, and activation of ECM-degrading enzymes including MMPs [78]. Competitive inhibition of urokinase-like plasminogen activator receptor (uPAR) binding to the ECM protein vitronectin (VN) by plasminogen activator inhibitor-1 (PAI-l) is capable of blocking adhesive sites and decreasing the adhesive strength of cells to their substratum. This can lead to increased cell detachment and migration [79]. Dissociation and motility of tumor cells is often associated with EMT wherein cells lose their epithelial characteristics and gain mesenchymal traits to achieve enhanced motility and invasive ability [80]. EMT may be a precondition for successful regulation and development of different types of cancer metastasis, as well as for normal cell differentiation during early development, and is therefore not specific to peritoneal dissemination. Factors involved in EMT have been extensively reviewed elsewhere [80]. Han et al. recently showed that in ovarian cancer, BTB and CNC homology 1 (BACH1), a basic leucine zipper transcription factor, promotes peritoneal metastasis by high mobility group AT-hook 2 (HMGA2)-mediated EMT [147]. E-cadherin, HGF/c-MET, ARL4C, integrins and other ECM protein/cell receptor interactors, and EMT-related proteins all contribute to cell detachment process in ovarian and gastric cancers.

In PDA, similar to ovarian and gastric cancer peritoneal metastasis, loss of E-cadherin enforces cell detachment and scattering by loosening cell-to-cell contacts and accelerating cell motility [148]. Restoration of E-cadherin expression reverses these processes indicating a direct role for this adhesion molecule in PDA peritoneal dissemination [148]. BACH1 promotes PDA peritoneal metastasis by repressing E-cadherin and enhancing EMT, particularly in cases driven by KRAS mutation [81]. ARL4C is significantly expressed in PDA and promotes growth and metastasis of this disease [83]. However, the role of this protein in PDA peritoneal metastasis has not been explored. Given its important role in cell detachment in ovarian and gastric cancer, investigation of ARL4C function in relation to PDA peritoneal metastasis is warranted. Similar to ovarian and gastric cancer, the EMT regulators HGF/c-Met have also been implicated in the peritoneal dissemination of PDA [149]. Connely and colleagues have shown that crizotinib, a small-molecule inhibitor of c-Met, suppresses HGF/c-Met signaling and RhoA activation thereby preventing peritoneal dissemination in PDA [149]. Others have also demonstrated favorable effects of crizotinib in pre-clinical models of PDA [84, 86]. This drug is currently being tested in PDA patients through the NCI MATCH trial (NCT02465060).

## Cell survival and transport in the peritoneal fluid

Once detached from the tumor mass, the cancer cells face several challenges to survive in the peritoneal fluid. In the absence of attachment to a substratum, the apoptosis of epithelial-derived cells is initiated. Apoptosis of anchorage-dependent cells due to detachment from ECM is called anoikis [82]. Tumor cells achieve anoikis resistance through inhibition of apoptosis mediators, adaptations in the cell metabolism, continuation of EMT status and oncogene activation. Several molecular factors engage in this process. Increased expression of survivin and X-linked inhibitor of apoptosis (XIAP), both members of the inhibitor of apoptosis protein (IAP) family suppress apoptosis by inhibiting caspases [150–152]. The free-floating cancer cells can also combat anoikis by remaining in the EMT state. This may be causatively linked with high expression of Smad-interacting protein 1 (Sip1), a two-handed zinc finger transcriptional repressor and a regulator of E-cadherin and MMP2 [87]. The microenvironment of the free abdominal space is hypoxic and deficient in glucose [153]. In response to this, HIF-1α, a master regulator of hypoxia, is induced in cancer cells. HIF-1α is reportedly involved in peritoneal dissemination in ovarian, gastric and colorectal cancers, as well as PDA [51, 153–155]. HIF-1α protein forms a heterodimer with the HIF-1β subunit then activates transcription of numerous target genes important in facilitating cell adaptation to the hypoxic environment [156]. In addition, HIF-1α induces EMT by activating transcription of the lysyl oxidase (LOX) family genes which encode secreted, copper-dependent amine oxidases [91]. Angiopoietin-like-4 (*ANGPTL4*), a secreted protein essential for tumor growth and resistance to anoikis in gastric cancer cells, is also upregulated by HIF-1α [112]. Inhibition of HIF-1α expression by dextran sulfate reduces gastric cancer peritoneal metastasis acting through decreased integrin β1 expression [90]. Intracellular signaling pathways such as PI3K/Akt, MEK/Erk and PTEN/PI3K/NF-κB/focal adhesion kinase (FAK) also play a role in cell survival and anoikis-resistance in gastric cancers [89, 113, 157–159]. FAK/Src signaling to the PI3-K/Akt-1 and MEK/Erk distinctively regulate cell survival and anoikis depending on the differentiation state of the cells [159]. Integrin ligation activates the NF-κB and PI3K/Akt pathways to upregulate pro-survival proteins B cell lymphoma 2 (Bcl-2) and FLICE-inhibitory protein (FLIP) thereby enhancing cell resistance to apoptosis while free floating [160]. FAK, a Src kinase, is a key integrin signaling molecule involved in the cell survival pathways which control EMT [113]. The tropomyosin-related kinase B (TrkB) is another potent pro-survival signal that renders epithelial cell resistance to caspase-associated anoikis. TrkB is a predictor for distant metastases and prognosis in gastric cancer [161]. Further, the chemokine-chemokine receptor CXCL12/CXCR4 pathway can induce EMT and affect cell survival through induction of the survival factor TNF-α [126, 162]. Activation of chemokine/TNF pathway is associated with peritoneal dissemination and anoikis resistance in multiple human cancers including ovarian and gastric [85, 88, 93, 94, 163, 164]. More details on chemokines in the context of metastasis are extensively reviewed elsewhere [163].

In addition to the above, one physical mechanism which facilitates malignant cell spread through the peritoneal cavity is the development of ascites. Ascites, as mentioned above, refers to the retention and accumulation of abnormal fluid in the peritoneal cavity, and frequently occurs in patients with peritoneal metastasis [50]. As disease progresses ascites volume tends to grow, and the diffuse flow of ascitic fluid facilitates the spread of detached cells to more distant sites in the abdomen and pelvis. The detached, floating malignant cells within the ascites can exist as single cells or as compact spheroid clusters. The latter retain tumor cell adhesive propensity for cell-cell adhesions of heterogeneous cell phenotypes within the clusters. Such adhesions protect spheroids against anoikis, and from elimination by intraperitoneal inflammatory cells [50]. It was shown *in vitro* in ovarian cancer that spheroids floating in ascites continue to express HGF and c-Met [114, 165]. Expression of these factors, in addition to regulating EMT, help retain the cell/ cluster’s future ability to adhere to mesothelial ECM *via* β_1_ integrins, a process that will be discussed in the next section [114, 165]. The spheroids may contain embedded circulating cancer-associated fibroblasts (CAFs) as well as activated mesothelial cells, both of which contribute to cancer cell resistance to anoikis by enabling metabolic support [100]. Tumor-associated macrophages (TAMs) embedded in spheroids promote early transcoelomic dissemination in ovarian cancer by generating immunosuppressive signals which protect the spheroids from immune attack by T cells [99]. Tumor cells in ascites differentially express epithelial cell adhesion molecule (EpCAM) on their surface [95]. Although EpCAM is naturally targeted by the immune system, how the cancer cells expressing this molecule evade this process and the extent and nature of the underlying immune responses remain undefined [95]. Mucin 16 (MUC16), the protein source of cancer antigen 125 (CA-125) facilitates peritoneal metastasis of ovarian cancer and contributes to the immune suppressive tumor microenvironment by inhibiting the activity of NK cells [95]. Additional studies will be required to further understand how tumor cells floating in isolation retain adhesion properties and combat immune surveillance. In summary, cancer cells require engagement of many cellular and molecular programs to survive flotation through the peritoneal cavity.

In PDA, the roles of anoikis resistance and EMT in IP metastasis are comparatively less delineated. Similar to ovarian and gastric cancers, activation of NF-κB pathway is implicated in PDA cells surviving anoikis through induction of antiapoptotic proteins such as cellular inhibitor of apoptotic protein-1 (CIAP-1) [96]. Overexpression of Lysyl oxidase-like 2 (LOXL2) enhances EMT-like process and increases migratory activity in PDA cells [97], whereas inhibition of FAK by a small-molecule inhibitor of tyrosine kinases FAK/PYK2 inhibits the same process in PDA cells [101]. Interaction between annexin II (ANX2), a calcium-dependent phospholipid-binding protein expressed on tumor cells [98], and the ECM protein tenascin C (TNC) contribute to EMT and anoikis resistance and stemness in PDA [166]. ANX2 was identified as a gemcitabine-resistant factor in PDA acting through Akt/mammalian target of rapamycin signaling pathway [102, 103]. The ANX2-TNC interaction is associated with peritoneal recurrence and poor outcomes following surgery in resected human primary PDA tissues and is implicated as a potential therapeutic target in PDA [166]. ANX2-TNC axis with respect to anoikis resistance has not been studied in ovarian and gastric cancer peritoneal metastasis. While a robust literature describes the process of gastric and ovarian cancer cell immune evasion during traverse of the peritoneal cavity, this topic has not been examined in PDA. Investigation of the immune evasion processes employed by PDAC is warranted.

## Attachment to the peritoneal mesothelial cells

The process of attaching to and developing metastatic deposits in a new organ is known as metastatic colonization. This step is considered the least efficient and most vulnerable phase in the whole process of metastasis [21]. In contrast with hematogenous spread where the endothelial wall forms the barrier, it is the PMC layer that forms the barrier in peritoneal metastatic colonization. Key for successful peritoneal colonization is the tumor cell attachment to the peritoneal mesothelium, a membrane composed of simple squamous epithelium. Peritoneum covers all surfaces in the gut, including organ surfaces, omentum, and the abdominal wall. An intact and functional mesothelial layer can inhibit attachment of cancer cells by secreting a mucus-like substance that cancer cells must penetrate in order to successfully reach attachment points on mesothelial cells. The intact mesothelial layer also defends against cancer cell penetration into the submesothelial space [53]. Damaged or senescent peritoneal mesothelium is more receptive to cancer cell adhesion than young or undamaged mesothelium in ovarian and gastric cancers [100]. Interaction between the mesothelial layer and ovarian/gastric cancer cells induces the mesothelial cells to secrete fibronectin [105, 106]. This occurs when TGF-β newly secreted by the cancer cells activates a TGF-β receptor 1 (TGF-βRI) RAC1/SMAD-mediated signaling pathway in the mesothelial cells that upregulates mesothelial cell fibronectin secretion. The secreted fibronectin helps tumor cells to attach to mesothelial cells, as the former have higher expression of the fibronectin receptor compared with other cells in the microenvironment [105, 106]. Mitra et al. have shown that siRNA or therapeutic antibody blockade of the cancer cell fibronectin binding partner α_5_β_1_-integrin inhibits c-Met/FAK/Src signaling and is therefore effective in both prevention and intervention settings [92]. Interestingly, the loss of E-cadherin, which facilitates detachment and resistance to anoikis as described in the earlier sections, is also coupled to reattachment at the distant metastatic site through induction of α_5_-integrin expression in cancer cells and increases in fibronectin production by mesothelial cells. This process occurs through the epithelial growth factor receptor (EGFR)/FAK/mitogen-activated protein kinase (MAPK) pathway [109]. Targeting α_5_-integrin could be a promising therapy for ovarian cancer peritoneal metastasis.

Another mechanism by which ovarian/gastric cancer cells adhere to the mesothelial surface is through the increased expression of extracellular proteases. When cancer cells come in contact with mesothelial cells, they stimulate the production of MMPs in mesothelial cells in a paracrine-like action. For example, MMP2 and MMP14 production is stimulated by the ovarian cancer cells while that of MMP1 is enhanced by the gastric cancer cells [110, 167]. Increased MMPs cleave the ECM proteins fibronectin and vitronectin present on the surface of the mesothelium into smaller fragments and enhance binding of the cancer cells to these ECMs through cancer cell α_5_β_1_-integrin and α_v_β_3_-integrin, respectively. The proteolytic activity of MMP2 against fibronectin and vitronectin also enhances adhesion of free-floating spheroids to the peritoneum [110]. Inhibition of MMP2 in ovarian cancer cells inhibited their adhesion to peritoneal surfaces in nude mice [110]. Increased MMP9 correlates with plasminogen-dependent degradation of the ECM and increased adhesion in malignant ovarian tissue and is facilitated by the endogenous *de novo*-expressed α_v_β_6_ integrin [168].

CD44H, a hyaluronic acid (HA) receptor, is another adhesion receptor that plays important role in the attachment of cancer cells to the peritoneal tissue [169]. CD44 is expressed by both cancer cells and peritoneal cells and can thus facilitate adhesion of CD44-binding ECM proteins HA and versican expressed on PMCs. Interaction between HA and CD44 mediates adhesion of cancer cells to PMCs and this interaction could potentially be targeted by pharmacologic intervention [19]. Ovarian cancer cells secrete exosomes enriched for CD44 to assist with attachment of cancer cells to HA expressed on mesothelial cells [170]. In gastric cancer, intraperitoneal CD44 mRNA levels of magnetically separated CD45-negative EpCAM-positive cells are indicative of peritoneal recurrence [108]. Further, mesothelial cells also secrete lysophosphatidic acid (LPA), which aids in mesothelial adhesion of ovarian cancer cells expressing receptors for LPA [171].

IL-1β/β1-integrin axis is implicated in ovarian cancer cell adhesion to mesothelial cells. IL-1β produced by ovarian cancer cells induces β1 expression on mesothelial cells facilitating adhesion. This axis plays a potential role in the attachment of accidentally dropped malignant cells following surgical resection which can account for some cases of peritoneal tumor recurrence in ovarian cancer [172]. ANX2 (mentioned previously) is produced by both ovarian cancer cells and peritoneal cells and its expression is regulated by ovarian cancer-peritoneal cell interactions promoting peritoneal dissemination. Inhibition of this protein by siRNA or by neutralizing antibodies significantly decreases PDA peritoneal metastasis [107].

Mesothelial intercellular adhesion molecule 1 (ICAM-1) and tumor cell CD43 (sialophorin) mediate adhesion between mesothelial and tumor cells in ovarian, colorectal and PDA cells [173]. However, in an *in vivo* study in gastric cancer ICAM-1 is indicated as a possible inhibitor of peritoneal metastasis due to ICAM-1/lymphocyte function-associated antigen 1 (LFA1)-mediated mononuclear cell recruitment [111]. These opposing findings raise uncertainty in considering ICAM-1 as a promising therapeutic target.

Heterotypic (cancer cell to mesothelial cell) interactions of mesothelin (MSLN) and its binding partner MUC16, which is the membrane-bound form of the ovarian cancer serum tumor marker CA-125 [104] are important for attachment of cancer cells to the mesothelial layer [174]. MSLN is a glycosylphosphatidyl inositol (GPI)-linked protein that is expressed by mesothelial cells lining the peritoneum [175] and highly overexpressed in various types of cancers, such as mesothelioma [176], ovarian cancer [176], gastric cancer [134], and PDA [115, 133]. MUC16 is overexpressed on the surface of ovarian cancer and PDA cells [115, 133] and is also present on the mesothelial cells lining the peritoneum. MSLN binding to MUC16 is dependent on the presence of an unidentified N-linked glycan structure on MUC16. MUC16-expressing ovarian cancer cells can bind specifically to the MSLN-expressing peritoneal lining to further peritoneal implantation [116]. In curatively resected stage III gastric cancer, MSLN expression is a significant predictive factor for peritoneal recurrence [134].

In PDA, as seen in ovarian and gastric cancers, senescent PMCs stimulate adhesion and migration of malignant cells [177]. Oxidative stress-dependent increase in ICAM-1 facilitates this process [178]. However, senescent peritoneal mesothelium fails to promote growth of PDA xenografts [177]. Similar to ovarian cancer, *in vitro* studies show that IL1-1β and TNF-α significantly stimulate adhesion of surgically dropped pancreatic tumor cells. It has been suggested that L-1β and TNF-α may account for tumor recurrence to the peritoneum following curative surgery [120]. Integrins, adhesion molecules and ECM proteins are also involved in peritoneal adhesion and subsequent colonization in PDA. Gene expression studies comparing optimized peritoneal disseminating cell lines with their parent pancreatic cancer cell line show increased induction of integrins α_3_, α_6_, and α_v_β_5_ and decreased expression of α_2_ integrin, CD44 antigen, and the principle HA receptor-derivatives hCD44H, and hCD44v 10 [42, 43]. However, data from Furuyama et al. [148] varies slightly from other reports. Although expression of adhesion molecule E-cadherin was changed in a pancreatic cancer cell line with high invasion and peritoneal dissemination, the process did not involve expression changes in other adhesion molecules such as CD44H and β_1_ [148]. Nevertheless, independent data by van Grevenstein et al. demonstrated an important role for increased CD44 and ICAM-1 adhesion molecules on mesothelial cells in facilitating cell-cell and cell-ECM interactions, solidifying a role for these proteins in cancer cell attachment to mesothelium [120].

## Cell invasion to the underlying connective tissue

Following ovarian cancer cell/spheroid attachment to the mesothelium, a process called mesothelial clearance occurs wherein mesothelial cells get pushed apart and retract. This facilitates cancer cell penetration/invasion [53]. More detailed descriptions on breaching of the mesothelial cell layer by malignant cells have been provided elsewhere [117]. Sites of mesothelial clearance are high in collagen-rich connective tissue matrices and these encourage interactions between cancer cells and submesothelial layer components such as myofibroblasts [117]. Ovarian cancer cells with a mesenchymal phenotype have a greater propensity for mesothelial clearance. The RAC1/SMAD signaling pathway induces a mesenchymal phenotype in the mesothelial cells [106]. Mesenchymally transitioned mesothelial cells can transform into a sizeable subpopulation of CAFs which help in subsequent mesothelial clearance by releasing a wide range of cytokines, growth factors, and ECM components [119]. HGF-soluble factor produced by peritoneal fibroblasts may set-up a congenial environment for peritoneal invasion and metastases by affecting the morphology of mesothelial cells [121]. Blockade of HGF/c-Met signaling system using an HGF antagonist NK4, when combined with intraperitoneal chemotherapy, suppressed gastric cancer peritoneal metastases in nude mice and enhanced survival [118]. NK4-mediated gene therapy also suppressed ovarian cancer peritoneal dissemination *in vivo* [122].

The production of MMPs and integrins is also important for promoting penetration into the submesothelial space. As cancer cells invade, the mesothelium stimulates secretion of MMP-1, MMP-2, and MMP-9 to induce mesothelial cell apoptosis [110, 167]. This is promoted by secretion of cancer cell Fas-ligand which then binds to a Fas receptor (CD 95) on mesothelial cells, and is regulated by a mesothelium-secreted protein, transglutaminase2 [179]. MMP9 secretion is enhanced by exosomes secreted by ovarian cancer cells enriched for CD44 [170]. MMP7, integrin α_3_β_1_ and its ligand laminin-5, a major ECM glycoprotein, also promote cell penetration into the submesothelial space [53, 124, 125]. Inhibition of this interaction using anti-integrin antibodies reduced the number of disseminated nodules in gastric cancer [124]. Annexin protein family member A1 (AnxA1) is a glucocorticoid-regulated anti-inflammatory protein associated with promotion of migration, invasion and peritoneal metastasis of gastric cancer cells. Elevated AnxA1 activates formyl peptide receptor signals, which subsequently activate the mitogen-activated protein kinase 1 (Erk)/β_1_-binding protein 1 pathway to promote invasiveness [180]. As mentioned earlier in the detachment section, BACH1 is also involved in the invasive progression of ovarian cancer at the peritoneal surface owing to its effect on EMT genes such as Snail family transcriptional repressor 2 (Slug) [147]. Another important player in cancer cell invasion is transmembrane-4-L6-family-1(TM4SF1), a four-transmembrane L6 family member. TM4SF1 regulates ovarian cancer cell invasion and migration by mediating cell motility and directional migration through filopodia formation [181].

In PDA, similar to ovarian and gastric cancers, loss of E-cadherin expression increases cell invasion *in vitro* and peritoneal dissemination *in vivo*, while restoration of E-cadherin expression reverses these processes [148]. As is ovarian cancer, BACH1 promotes PDA cell migration and invasion in part by repressing E-cadherin expression [81]. MSLN and MUC16 co-overexpression and mutual binding of MSLN to MUC16 markedly enhances PDA cell migration and invasion. This involves the selective induction of MMP7 and occurs *via* a p38 MAPK-dependent pathway [182]. In addition, we have recently shown that MSLN promotes peritoneal carcinomatosis of PDA by positively regulating several processes including cancer cell invasion [183]. TM4SF1 also appears to be broadly involved in the cancer-to-mesothelial cell attachment process independent of histology. In PDA, TM4SF1 on the cell surface collaborates with DDR1 to increase the formation of invadopodia and the expression of MMP2 and MMP9 to regulate invasion and migration [184]. Eukaryotic elongation factor 1 α2 (eEF1A2) significantly promotes the migration and invasion of PDA cells and their IP metastatic ability by upregulating MMP9 through Akt activation [123]. Involvement of Akt has also been indicated in gastric cancer as mentioned above [185]. More work will be required to delineate additional molecular mediators of PDA invasion through the mesothelial layer.

## Establishment of metastatic deposits

Once tumor cells have adhered to and penetrated the mesothelial cell layer, the connective tissue under the mesothelium provides a protective microenvironment (niche) for seeding cancer nodules and assisting their growth [53, 186]. In addition, mesothelial cells may also create a novel tissue niche that facilitates gastric cancer invasion, suggesting stabilization of PMCs as a potentially effective therapy for the prevention of peritoneal invasion in gastric cancer [125]. Cancer cell interactions with the mesothelial cells covering the surface of the omentum involve the tumor suppressor micro-RNA miR-193b, a translational regulator that affects peritoneal metastatic deposition in ovarian cancer. Downregulation of miR-193b in ovarian cancer cells induces increased expression of urokinase-type plasminogen activator, a known tumor-associated protease, which enables metastasizing cancer cells to proliferate and colonize [187]. Several miRNAs are dysregulated and play a role in the peritoneal metastasis of gastric cancer [188]. Mechanisms of interaction between peritoneally metastasizing cancer cells and the omentum seem to be common in ovarian and gastric cancers, as well as in PDA [127, 189, 190]. This convergence attests to a conserved symbiotic relation between cancer cells and mesothelium. Further understanding of the underlying mechanisms may help researchers discover new targets for therapy.

The successfully adapted cancer cells reprogram the microenvironment to form an “activated tumor stroma” in the niche. This stroma is very heterogeneous, consisting of cellular and acellular components including CAFs, endothelial cells, immune cells, and modified ECMs. Soluble proteins such as cytokines and growth factors are also enriched. All the above components promote colony formation and proliferation/growth at the metastatic site [21]. The metastatic reprogramming happens through extensive reciprocal interactions between the cancer cells and the other cell types within the metastatic niche [21].

Stromal myofibroblasts play a central role in pathogenesis of peritoneal fibrosis induced by cancer cells during metastatic colonization. It is hypothesized that this fibrosis provides a congenial environment for peritoneal metastasic deposits in gastric cancer [130]. In ovarian cancer, myofibroblasts in omentum are activated by tumor cells to promote metastatic deposition, and tumor cell growth, adhesion and invasiveness [191]. Cancer cells induce reprogramming of resident normal fibroblasts in the peritoneal basement membrane to a CAF phenotype. This is attained through the decreased expression of miR-214 and miR-31, and increased expression of miR-155, all targeting the chemokine mediator CCL5 [192]. The miRNAs act as mediators between cancer cells and the tumor microenvironment and are regulated by environmental paracrine signals [136]. PDA is one of the most stroma-rich cancers and is characterized by excessive desmoplasia which plays a crucial role in its aggressive behavior [193]. Few studies, however, have assessed cancer-stromal cell interactions at peritoneally disseminated sites, although this could constitute a new therapeutic target to prevent the peritoneal dissemination of PDA [121]. Myofibroblasts in the stroma of PDA promote tumor proliferation, invasion and metastasis by increasing ECM and secretion of several growth factors. However, the role of myofibroblasts in PDA is controversial. Akagawa et al. showed that at peritoneally disseminated sites of PDA peritoneal myofibroblasts that are positive for smooth muscle actin (αSMA+) promote dissemination and thus may be a potential new therapeutic target [121]. In line with this, others have also shown that stromal CAFs modulate PDA cells to attain aggressive phenotypes, i.e., invasive EMT and proliferative types [194]. However, it is unclear whether αSMA+ CAF targeting strategy would have an overall benefit for patients. Transgenic knockout mice ablated of subsets of α-SMA+ myofibroblasts have more aggressive primary PDA [195]. This corresponded with suppressed immune surveillance and diminished survival. Similarly, PDA patients with a lower myofibroblast percentage in tumors had reduced survival [195]. Targeting of SMA+ CAFs appears to be a risky strategy for improving PDA patient outcomes even if it could be effective against peritoneal metastasis formation.

In the tumor microenvironment, cancer cells also directly interact with immune cells. In ovarian cancer, immune cells such as M2 macrophage-like TAMs secrete EGF, which upregulates α_M_β_2_ integrin on TAMs and ICAM-1 on tumor cells to promote association between tumor cells and TAMs. EGF secreted by TAMs activates EGFR on tumor cells, which in turn upregulates VEGF signaling in surrounding tumor cells to support tumor cell proliferation and migration [99]. Ly6G+ CD11b+ cells stimulate PDA cell proliferation and hepatic metastasis, while their depletion reduces the same. This effect is sensitive to pharmacological inhibition of MEK and Hedgehog [128]. It remains to be seen if MEK immune cell proliferation axis may also be applicable to peritoneal metastasis of PDA. Engle et al. [129] recently showed that carbohydrate antigen 19-9 (CA19-9)-mediated pancreatitis-induced proliferative changes accelerated PDA peritoneal metastasis in mice through hyperactivation of EGFR signaling. In this case, forced expression of CA19-9 on murine cancer cells, which typically lack the enzymes required to produce this human tumor marker, led to pancreatitis through recruitment of inflammatory monocytes and macrophages. Interactions between cancer cells and immune cells need to be explored in more detail in the context of peritoneal metastasis.

Cancer cells can also induce metabolic reprogramming of the omental adipocytes to stimulate lipolysis. The adipocytes in turn induce the expression of fatty acid-binding protein 4 (FABP4), a fatty acid transporter in the cancer cells, which facilitates efficient cancer cell uptake of free fatty acids (FFAs) released by the adipocytes. Cancer cells can utilize these FFAs as a source of nutrition to drive tumor growth [196]. Omental adipocytes enhance the invasiveness of gastric cancer cells by oleic acid-induced activation of PI3K-Akt signaling pathway involving associated upregulation of the key pro-invasion factor MMP2 [185]. Similar to ovarian and gastric cancers, omental fat-secreted factors may also play a role in PDA peritoneal spread. These factors increase expression of several transcription factors, ECM proteins, and adhesion molecules and may therefore increase several metastatic processes such as growth, migration, invasion, and chemoresistance in PDA. Adipose tissue-derived stem cells also promote PDA cell proliferation and invasion [127]. They do this potentially through SDF-1/CXCR4 axis [197]. Sphingosine-1-phosphate (S1P) is a bioactive lipid mediator generated by sphingosine kinases, SphK1, and SphK2. S1P regulates cell proliferation, invasion, and angiogenesis in cancer cells [198, 199]. S1P generated by SphK1 in the host microenvironment promotes PDA peritoneal carcinomatosis by stimulating proliferation of cancer cells and reducing inflammatory cell infiltrate [200]. There are currently no therapeutics targeting interactions of adipocytes and cancer cells.

Immediately following metastatic colonization, establishment of new microvasculature or neoangiogenesisis is required for the proliferation and growth of the new metastases [50]. Several pro-angiogenic factors and their corresponding receptors are involved in this process [201]. VEGF is a well-known multifunctional pro-angiogenic cellular factor which can induce neovascularization [131, 132]. Integrin β_3_ and VEGF expression correlate and can synergistically enhance tumor angiogenesis to play a crucial role in metastasis of gastric carcinoma [202]. In human surgical specimens from patients with stage II gastric cancer with serosal invasion, VEGF levels correlated with peritoneal metastasis, and so VEGF may be a useful indicator of peritoneal recurrence [137]. Other angiogenic factors such as platelet-derived growth factor (PDGF), fibroblast growth factors (FGFs), and angiopoietins have also been identified to assist in the establishment of new microvasculature [201]. FGF is an important regulator of angiogenic factors where FGF-1 and FGF-2 function through the activation of the Akt signaling pathway [138]. In addition to tumor cells, subsets of hypoxic CD105-expressing mesothelial cells are possible sources of FGFs as well as VEGF [203]. FGF can further augment synthesis of VEGF [204]. Angiopoietins are critical for vessel maturation and participate in migration, adhesion and survival of endothelial cells. Four different angiopoetins (Ang-1, Ang-2, Ang-3, Ang-4) have been described, all of them binding to the tyrosine kinase receptor tie-2 [135, 205]. Microenvironment-derived dendritic cell subsets may differentially regulate angiogenesis in ovarian cancer. Plasmacytoid dendritic cells (PDCs) and stromal-derived factor (SDF-1/CXCL-12) are involved where PDCs induce angiogenesis through production of TNF-α and IL-8 [206]. Proteases are also important in the angiogenic process. MMP1-protease-activated receptor-1 (PAR1)-CXCR1/2 paracrine pathways have been suggested as new targets for ovarian cancer therapy involving peritoneal metastasis. PAR-1 is a G protein-coupled receptor demonstrated to be an important signal transducer of angiogenesis and metastasis in an ovarian cancer mouse model of peritoneal dissemination. PAR1-1 is activated by MMP1 and this activation induces the secretion of angiogenic factors IL-8 and growth-regulated oncogene-α (GRO-α) from ovarian carcinoma cells, which act on endothelial CXCR1/2 receptors in a paracrine manner, leading to endothelial cell proliferation, tube formation and migration [207]. PAR-1 expression is also correlated with peritoneal dissemination in gastric cancer [208]. There is limited information about factors controlling neoangiogenesis during peritoneal colonization in PDA. Our recent study indicated a functional role for MSLN expressed on PDA cells to induce blood vessel formation during metastatic colonization [183]. Consistent with our study, Mizukami et al. [209] demonstrated that MSLN KO or blockade with the clinical anti-MSLN monoclonal antibody amatuximab can delay intraperitoneal tumor growth in the presence or absence of intact immune signaling. Importantly, metastasic peritoneal deposits are still established in treated mice, albeit more slowly, which may limit the therapeutic potential of MSLN blockade in this setting. Vasohibin-2 (VASH2) is an endothelium-derived angiogenic factor expressed in cancer cells that promotes tumor growth and peritoneal dissemination in ovarian cancer. VASH2 stimulation of angiogenesis is related to decrease of mir-200b [210]. Like ovarian cancer, PDA expresses VASH2 [211]. VASH2 expression is associated with poor prognosis in PDA patients [212]. VASH2 effects both PDA cells and the tumor microenvironment by promoting tubulin detyrosination-lead tumor cell migration, tumor angiogenesis, as well as induction of myeloid derived suppressor cells [211]. Many anti-angiogenic agents are approved or under clinical development for patients with ovarian cancer. These include mAb directed against VEGFA, protein-based agents that neutralize all isoforms of VEGF and angiopoietins 1 and 2, as well as small molecule tyrosine kinase inhibitors [19]. Targeting VEGF is also considered an attractive strategy to inhibit peritoneal dissemination in gastric cancer. VEGF receptor antisense therapy has recently been shown to inhibit angiogenesis in this tumor [213]. Thus far, anti-angiogenic therapy has produced no benefit for patients with PDA.

Interactions between cancer cells and the microenvironment during the metastatic colonization process are complemented by other signaling events. HGF/c-Met signaling is implicated in cell proliferation and invasion in PDA peritoneal metastasis [149]. Similar to ovarian and gastric cancers as mentioned above in invasion section, blockade of the HGF receptor pathway by administration of recombinant NK4, a four-kringle fragment of HGF which functions as its antagonist, inhibits growth, invasion, and distant metastasis of orthotopically implanted PDA cells. NK4 prolongs invasion of cells into the peritoneal wall, suppresses peritoneal dissemination, ascites accumulation and increases survival of mice [214]. Antitumor effects of NK4 also include its antiangiogenic actions [214]. Simultaneous targeting of both tumor angiogenesis and the HGF-mediated invasion and metastasis could form a new approach to treating patients with PDA peritoneal metastasis. Tyrosine kinases FAK and PYK2 are activated and expressed in human PDA cell lines, patient-derived PDA tumors as well as in stromal components. Inhibition of these kinases by PF-562,271 inhibits PDA peritoneal metastasis collaterally altering the tumor microenvironment [101].

Interactions between the cancer cells and the microenvironment are important in the peritoneal implantation of ovarian and gastric cancer cells, as well as in PDA. However, the initial cross talk between the cancer cells and the microenvironment occurs within a small window of time and the complexity of the interactions makes comprehensive understanding of the process a challenge. Because interruption of this crosstalk may constitute a therapeutic vulnerability in the process of peritoneal metastasis, further research in this area should be pursued.

## Concluding remarks

We have outlined the biological mechanisms that contribute to peritoneal metastasis of PDA with comparison with mechanisms observed for ovarian and gastric cancers. The molecular pathways that contribute to peritoneal metastasis are distinct from those that cause hematogenous spread. Peritoneal metastasis occurs in discrete steps that are preserved across tumor types. While the identity of some molecules contributing to these steps varies across tumor types, the classes of molecules required remain the same. For instance, while the anti-apoptotic proteins survivin and XIAP confer anoikis resistance in ovarian cancer, the related molecule CIAP-1 plays this role in PDA. The pathway similarities among different tumor histologies suggest that cancer cells co-opt pre-existing programs used for normal physiologic processes to further peritoneal metastasis.

Some molecules participate in multiple steps of the peritoneal metastatic cascade. For example, HGF/Met pathway plays a role in cell detachment and also in invasion and metastatic colonization steps. Likewise, integrins and MMPs participate in cell adherence, invasion, and metastatic deposit formation. A few of these molecules may play opposing roles in the different steps, acting as augmenters of metastasis in one process and inhibitors in another. E-cadherin expression inhibits exfoliation of cancer cells from the primary tumor and also takes part in re-attachment of cancer cells to the mesothelial surface when they reach the peritoneum. Of note, many molecular mediators that have been well researched in ovarian and gastric models have not been examined at all in PDA.

Peritoneal dissemination remains an important and understudied problem in pancreatic cancer. Additional work will be required to better delineate the specific molecular mediators used by PDA to metastasize to the peritoneum. A better understanding of these details may facilitate the design of therapeutic strategies useful in the clinical setting.
